# Electrical synapses for a pooling layer of the convolutional neural network in retinas

**DOI:** 10.3389/fncel.2023.1281786

**Published:** 2023-10-30

**Authors:** Yoshihiko Tsukamoto

**Affiliations:** ^1^Department of Biology, Hyogo Medical University, Nishinomiya, Hyogo, Japan; ^2^Studio EM-Retina, Satonaka, Nishinomiya, Hyogo, Japan; ^3^Center for Systems Vision Science, Organization of Science and Technology, Ritsumeikan University, Kusatsu, Shiga, Japan

**Keywords:** gap junctions, ribbon synapses, electron microscopy, connectome, retinal neurocircuitry, rod pathway

## Abstract

We have an example of a synergetic effect between neuroscience and connectome via artificial intelligence. The invention of Neocognitron, a machine learning algorithm, was inspired by the visual cortical circuitry for complex cells to be made by combinations of simple cells, which uses a hierarchical convolutional neural network (CNN). The CNN machine learning algorithm is powerful in classifying neuron borderlines on electron micrograph images for automatized connectomic analysis. CNN is also useful as a functional framework to analyze the neurocircuitry of the visual system. The visual system encodes visual patterns in the retina and decodes them in the corresponding cortical areas. The knowledge of evolutionarily chosen mechanisms in retinas may help the innovation of new algorithms. Since over a half-century ago, a classical style of serial section transmission electron microscopy has vastly contributed to cell biology. It is still useful to comprehensively analyze the small area of retinal neurocircuitry that is rich in natural intelligence of pattern recognition. I discuss the perspective of our study on the primary rod signal pathway in mouse and macaque retinas with special reference to electrical synapses. Photon detection under the scotopic condition needs absolute sensitivity but no intricate pattern recognition. This extreme case is regarded as the most simplified pattern recognition of the input with no autocorrelation. A comparative study of mouse and macaque retinas, where exists the 7-fold difference in linear size, may give us the underlying principle with quantitative verification of their adaptational designs of neurocircuitry.

## 1. Introduction

Electron microscopy resolved the controversy between Cajal’s neuron theory and Golgi’s reticular theory by observing synaptic clefts between two nerve cells. However, the situation is not as simple as imagined. There are wide and narrow types of synaptic clefts: approximately 20 nm and 2 nm. Each neuron is bounded by its own cell membrane and has specialized organelles for contact communication: chemical and electrical synapses. Chemical synapses have wide clefts, while electrical synapses have narrow clefts. Cajal predicted the existence of chemical synapses which are characterized by unidirectional flow of information and connectional specificity. But he rejected the hypothesis of intercellular coupling. In his time, light microscopy could not identify gap junctions. The electrical synapses are characterized by the bidirectional flow of electricity and cytoplasmic continuity via connexons. If Cajal had modestly reserved his judgment on the intercellular coupling, his prediction could have become even more satisfactory (Cowan and Kandel, 2001; Sterling and Laughlin, 2015).

**Figure 1:**
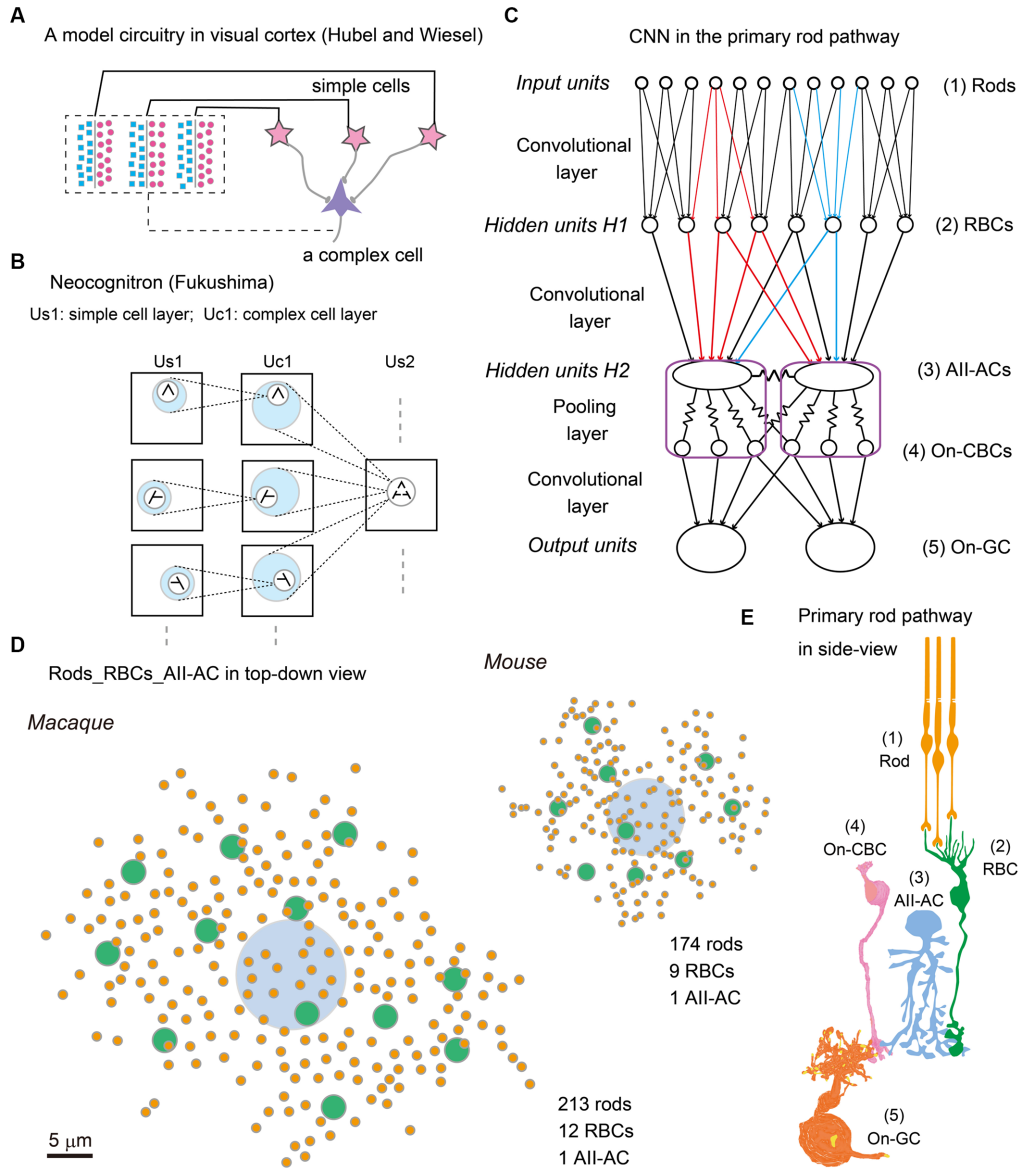
**(A)** Convolutional neural network (CNN), (Redrawn from Hubel and Wiesel, 1962) on the model circuitry for producing complex cells from simple cells in the cat visual cortex. **(B)** Neocognitron (Redrawn from Fukushima, 1980, 1988), a machine learning algorithm inspired by the above CNN model, for pattern recognition with lateral shift-invariance. **(C)** CNN applied to the primary rod pathway in the retina. Each rod in the first layer (*Input units*) sends photon capture signals to 1 ~ 3 rod bipolar cells (RBCs) at the second layer (*Hidden units H1*). Each RBC collects signals from 25 ~ 30 rods, reduces them into a single value, and diverges it to 10 ~ 20 AII–amacrine cells (AII–ACs). AII–ACs and on cone bipolar cells (On-CBCs) (*Hidden units H2*) are mutually coupled through gap junctions to form a syncytial network. On ganglion cells (On-GCs) (*Output units*) send signals to the central nervous system. **(D)** Top-down view illustration of three-layered networks from rods (orange) to RBCs (green) to AII-ACs (light blue) in macaque and mouse retinas. **(E)** Side view illustration of the primary rod pathway: (1) Rods, (2) RBCs, (3) AII–ACs, (4) On-CBCs, and (5) On–GCs.

After half a century since then, two new waves have emerged, deep learning and connectome. Deep learning is software for pattern recognition with a hierarchical architecture of the convolutional neural network (CNN) empowered by an end-to-end supervised-learning algorithm called backpropagation (LeCun et al., 1989, 2015). Connectome is a complex of hardware and software for the automatization of efficiently making serial sections for scanning electron microscopy and image processing for 3D reconstruction of neurocircuitry (Seung, 2012). Hubel and Wiesel explained how a complex cell is generated by a combination of simple cells (Figure 1A) by using a model of the CNN of the visual cortical circuitry (Hubel and Wiesel, 1962). Being inspired by those physiological findings, Fukushima made a new algorithm called Neocognitron for pattern recognition, which has a hierarchical architecture of CNN and consequently a unique function not affected by deformations and shifts in the position of input patterns (Figure 1B) (Fukushima, 1980, 1988). The CNN machine learning algorithm used by current connectome software is powerful for pattern recognition to classify neuron contours on electron micrographic images. This exemplifies a synergetic effect between neuroscience and connectome via artificial intelligence.

A classical style of serial section transmission electron microscopy (SSTEM) has contributed to cell biology for over a half-century. The classical SSTEM we use is still useful to comprehensively analyze the small area of retinal neurocircuitry that is rich in natural intelligence of pattern recognition. Here, I discuss the perspectives of our study on the neuronal circuitry in mouse and monkey retinas with special reference to gap junctions. We need some functional framework for interpreting the structural data. So, my first question is whether CNN is applicable to retinal circuits as a functional framework. Using the hypothetical CNN framework, the next question is how structural parameters are related to the information-processing functions of neuronal circuitry.

## 2. Application of CNN motifs to retinal circuitry

The layer of CNN is another name for the layer comprised of receptive fields (RFs) from the architectural point of view. The RF of a neuron in the visual system can be defined as the area of the retina from which the activity of a neuron can be influenced by light (Nicholls et al., 2001). For example, the activity of a ganglion cell (GC) in the retina or a cortical cell in the visual area shows an increase or decrease in the firing rate only when illumination is changed over a restricted area of the retina known as an RF. The RF is characterized by the sensitivity gradient profile that is generally high at the center and becomes lower toward the surround. The RFs of several GCs partially overlap such that every point in intermediate space is covered almost equally (Borghuis et al., 2008). The retinal GCs encode visual signals, which are decoded through the corresponding areas in the central nervous system with a hierarchical architecture of CNN. Both encoding and decoding systems must be closely reflected on each other in some orchestrating way.

Is CNN applicable to the connectomic study of retinal neural networks? At first glance, supervised-learning algorithms such as backpropagation seem irrelevant to the retinal circuitry because retinal circuitry is neither involved in learning nor furnishing such neuronal feedback mechanisms. Nevertheless, the retina shows various adaptive circuitry designs from the evolutionary perspective. So, if we reserve the yet unknown but possible alternative mechanisms for the feedback part of the deep learning scheme, its forward part seems to be applicable. Next, the retinal circuitry differs from most central nervous systems in that only GCs evoke typical action potentials. Other retinal cells, rod and cone photoreceptors, bipolar cells (BCs), and horizontal cells except amacrine cells usually operate by means of graded potentials. This is simply because long-distance communications are rarely required within the retina, just fitting to a principle of economical wiring (Sterling and Laughlin, 2015). Finally, according to the current CNN model by LeCun et al. (2015), a typical neuron has three key processes: ① weighted reception, ② pooling, and ③ nonlinear activation. All these processes are adopted by the linear–nonlinear (LN) model of retinal neurons that combines ① a linearly weighted sum (or ② pooling) and ③ an instantaneous (or static) nonlinearity function (Sakai et al., 1995; Chander and Chichilnisky, 2001; Chichilnisky, 2001; Kim and Rieke, 2001). In addition, the CNN model has four key architectural motifs: ① local connections, ② shared weights, ③ pooling, and ④ multiple layers. Before item-by-item checking, we brief the rod pathway in the next.

## 3. How signal-to-noise ratio is conditioned by structural parameters

Barlow once suggested a criterion that may help in establishing a candidate signal as a really utilized neurophysiological code in a work session of Neuroscience Research Program (Perkel and Bullock, 1968). That criterion is the efficiency measured as signal-to-noise ratio (S/N) in the neural output compared to the S/N of the input. Researchers may postulate more than one candidate which are all plausible from the statistical point of view. If one of them is obviously subject to the evolutional adaptation to the surrounding conditions in their structural architecture, that candidate code may be regarded as the true one. This requires clarifying what neuronal (input–to–output) connections and what associated structural parameters are closely related to improving the S/N. The varying number of synaptic contacts and the areal range of their summation are both closely related to the S/N improvement in the CNN scheme.

Figure 1C illustrates the CNN model of the primary rod signal pathway which is dealt with in this article. The first input units are rod photoreceptors, and the first hidden units (H1) are rod bipolar cells (RBCs) (Figure 1E). Both units are connected by a convolutional layer. But the unique feature of this primary rod pathway is that the second hidden units (H2) consist of a complex of AII amacrine cells and On-cone bipolar cells (On-CBCs), which are directly or indirectly coupled together through gap junctions, forming a syncytium. The resultant pooled signals are relayed to output units GCs by chemical synapses and then GC signals are emitted to the central nervous system.

Figure 1D shows the distribution of rod spherules (green) and RBCs (orange) whose circuitry makes their signals destined for an AII amacrine cell (light blue) in mouse and macaque retinas (Tsukamoto and Omi, 2013 for mouse; unpublished data for macaque). More numerous rods and RBCs converge to a downstream AII amacrine cell, and the rod-collecting area of an AII amacrine cell is linearly 2-fold greater, in the macaque retina than in the mouse retina. These differences may be related to their eye sizes. The length encompassed by 1° of visual angle on the retinal surface is approximately 30 μm in mice and 210 μm in macaque monkeys, resulting in a ratio of 1:7. As this size increases, the image resolution per unit length becomes higher (7-fold) but the photon density decreases. Therefore, the larger rod-collecting area in the macaque retina still retains high resolution (3.5-fold) by increasing the number of collected rods (1.2-fold) in comparison with the mouse retina. These numerical differences are furthermore reflected in the synaptic weightings at other parts of this primary rod pathway. The wider space is capable of harnessing more powerful signal transmission devices: more synaptic contacts and larger synaptic areas. The number of RB invaginating dendrites into a rod spherule is 1.2-fold greater (2.4 versus 2 dendrites/rod), and the synaptic contact area between RB invaginating dendrites and the rod spherule membrane is 1.8-fold greater (2.5 versus 1.4 μm^2^/rod), in the macaque retina than the mouse retina (Tsukamoto and Omi, 2022). Thus, we can quantify the differences in the adaptation to the different sizes of optical images by comparing mouse and macaque retinas.

## 4. How CNN is compatible with the rod pathway

The key neuronal processes [N_] and architectural motifs [A_] of CNN are compared to the properties of the rod pathway as follows.

**Figure 2:**
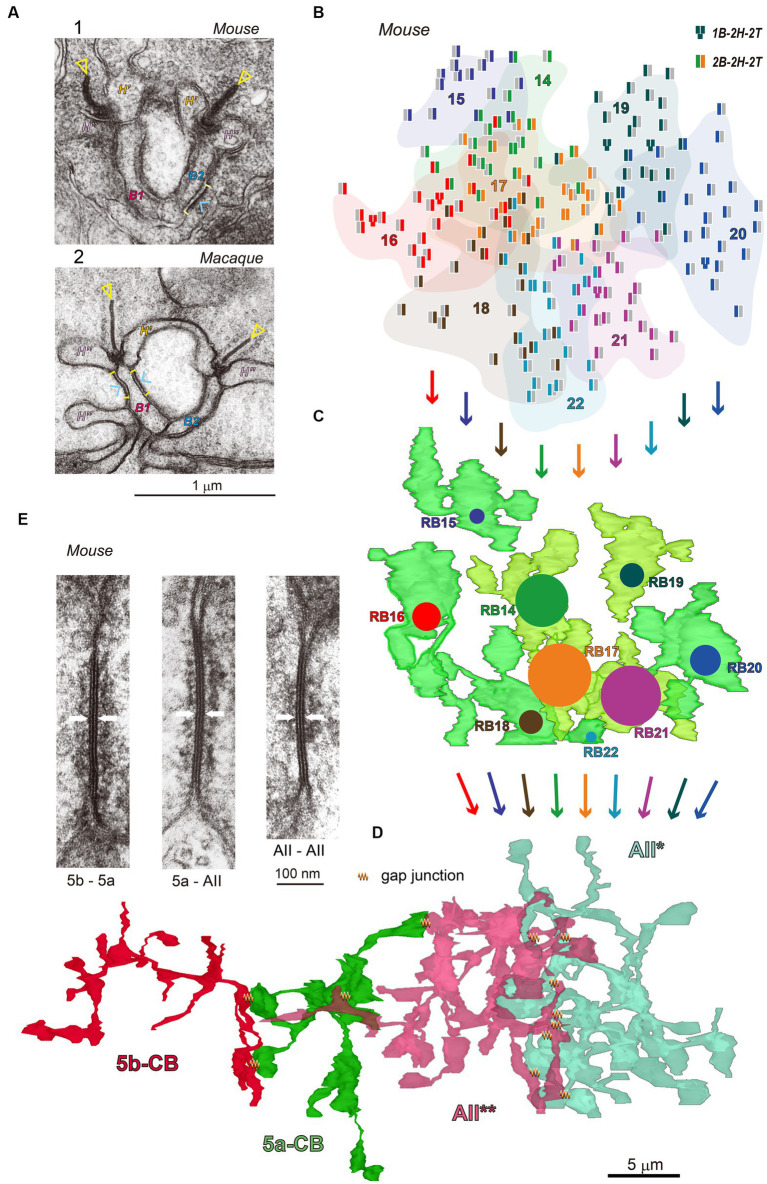
**(A)** Electron micrographs of ribbon synapses in mouse (A-1) and macaque (A-2) rod terminals where rods release glutamate by vesicular exocytosis toward RB dendrites and horizontal cell (HC) processes. Two ribbons (triangles) are each in front of an RB dendrite (B1 or B2) and flanked by two horizontal cell processes (H′ and H″). The rod cell membrane sites (brackets) facing the RB dendrites are endowed with fluffy presynaptic density (arrowheads). This subcellular web substantiates that rod–RB contact sites are rich in trans-synaptic molecules. **(B–D)** Illustrations of a pathway of rods **(B)**, RBCs **(C)**, and AII* amacrine cell **(D)** in the top-down view in the mouse retina. A total of 174 rods are collected via 9 RBCs (B14–B22) which are summed at AII* amacrine cell with different weights (as depicted by colored circular areas). Neighboring AII* and AII** amacrine cells are mutually coupled via gap junctions. **(E)** Electron micrographs of gap junctions (arrows) between (1) two AII amacrine cells, (2) Type 5a On-CBC and AII amacrine cell, and (3) Type 5b and 5a On-CBCs in the mouse retina. Cytoskeletal fibrous materials are found in the subsurface cytoplasm.

[A_④ Multiple layers: Rod pathway] Photon detection is challenging for mouse vision under starlit night conditions. To ensure this, a neural circuit for S/N improvement is implemented in the rod–rod bipolar–AII amacrine pathway. This pathway exemplifies how structural data can help with analysis. Figure 2 provides an overview created by editing the previously published data (Tsukamoto and Omi, 2013, 2017, 2022). Figure 2A compares electron microscopy images of mouse and macaque Rod–RB synapses. Figure 2B shows the distribution of rod synaptic terminals in the mouse retina. Those rod outputs transmit via nine RBCs (Figure 2C) to AII* amacrine cell (Figure 2D). Figures 2D,E present the locations and electron microscopy images of gap junctions among AII amacrine cells (AII* and AII**) and type-identified On-CBCs (5a and 5b).
[N_③ Nonlinear activation: Rod–RBC] The rod–to–RBC synapses perform the thresholding transform to reduce noises (Field and Rieke, 2002). In most cases, the number of outputs from a rod terminal is two.
[N_① Weighted reception: RBC] An RBC receives ~25 weighted rod signals. The weight coefficients are mostly one but in a few cases two, where one RBC connects with one rod through two invaginating dendrites.
[N_② Pooling: RBC] An RBC pools many rod signals through the dendritic arbor and produces a representative output signal. Under starlit night conditions, each rod rarely absorbs a photon; this convergent pathway is thought to increase the probability of photon absorption. Under mesopic light conditions, however, many rods absorb one or more photons simultaneously; this convergent pathway is thought to convey the graded light response.
[N_③ Nonlinear activation: RBC–AII] The signal transfer from an RBC to an AII amacrine cell is well described using linear–nonlinear cascade model (Jarsky et al., 2011).
[A_① Local connections: RBCs] In mice, ~25 rods in the vicinity contact with one RBC through ribbon synapses.
[A_② Shared weights: RBCs] Neighboring RBCs have similar lateral strides and dendrites, thus they receive similar numbers (~25) of rod inputs.
[N_① Weighted reception: AII*] The number of RBC–AII* synapses varies greatly among RBCs. The central three RBCs have many contacts (24 to 34), while the surrounding six RBCs have few contacts (1 to 8) as depicted by colored circles (Figure 2C).
[N_② Pooling: AII] Electrical potential spreads electrotonically for averaging within the AII amacrine cell.
[A_① Local connections: AIIs] In mice, ~10 RBCs in the vicinity contact with one AII amacrine cell through ribbon synapses.
[A_② Shared weights: AIIs] Three neighboring AII amacrine cells had the similar distribution profiles of the number of contacts with 9 to 12 RB axon terminals (Tsukamoto and Omi, 2013).
[N_③ Nonlinear activation: AII–AII–On-CBC] AII amacrine cells are electrically coupled to other AII amacrine cells and On-CBC. Electrical transmission is linear, but its efficacy depends on frequency components. Lower frequencies are more transferable than higher frequencies.
[A_③ Pooling: AII–AII–On-CBC] The pooling area is large. AII amacrine cells in the neighboring area make a syncytial network via gap junctions. This coupling extends to On-CBCs as well (Figures 2D,E). However, Tian et al. showed that AII amacrine cells were coupled electrically but Na channel-mediated effects on EPSPs appeared to occur at the single-cell rather than the AII network level (Tian et al., 2010). The Na channel-mediated acceleration may occur in rather restricted areas (Tamalu and Watanabe, 2007).
[N_③ Nonlinear activation: On-CBC–GC] On-CBC nonlinearly activates GC (Field et al., 2009).

## 5. EM methodology for 3D observation

Every methodology has its own pros and cons. Our relatively thick (~90 nm) sections for SSTEM (at 80 kV) have two major (i, ii) and two minor (iii, iv) advantages. (i) Thicker sections contain richer supramolecular constituents whose spatial relationship remains intact within that thickness. Micrographic images with rich cytological contexts provide many clues that help human pattern recognition to solve jigsaw puzzles for 3D circuitry reconstruction, (ii) Tilting and reimaging the sections at higher magnifications ensures that electrical synaptic contacts are identified as gap junctions, (iii) The number of sections required to cover the same sample volume decreases inversely with thickness. This idea is not extraordinary because high-voltage electron microscopy pursues this merit to observe the volume of a sample efficiently, and (iv) Using the thick sections is safer for making serial sections without loss.

As an example of (i), Figure 2A shows the rod spherule ribbon synaptic complexes in the mouse (A-1) and the macaque (A-2). Presynaptic fluffy density indicates the structural marker of molecular complexes involved in synaptic transmission (Ueda et al., 1997; Martemyanov and Sampath, 2017). As an example of (ii), Figure 2E shows gap junctions between three different couples of On-CBCs and AII amacrine cells (Tsukamoto and Omi, 2017). The filamentous density beneath the surface indicates cytoskeletal architecture. Our classic SSTEM has several advantages but requires long hours of human labor. Therefore, we limit our samples to only small areas, pieces of retinas.

To overcome the limitations of the classic EM, two new EM physical procedures have been in progress: Serial block-face scanning electron microscopy (SBSEM) (Denk and Horstmann, 2004) and the automated tape-collecting ultramicrotome (ATUM) (Hayworth et al., 2014). One of the advantages of SBSEM is that photographing the block faces physically assures the alignment between successive images. SBSEM facilitates automatic image processing, but reimaging is impossible. ATUM preserves the ultrathin sections that are collected from the knife’s water boat on a conveyor belt made of plastic tape. The SEM images of the surface of sections are gathered for surveillance at low resolution. Then, the regions of interest are reimaged at high resolution. In conjunction with these hardware developments, many levels of software are created for automatic operations, such as KNOSSOS (Helmstaedter et al., 2011), Fiji (Schindelin et al., 2012), TrakEM2 (Cardona et al., 2012), CATMAID (Witvliet et al., 2021), and VAST (Berger et al., 2018). Notably, another example of the synergetic effect between neuroscience and the machine learning algorithm of CNN is demonstrated for detecting global contours (Chen et al., 2014; Lee et al., 2015).

## 6. Circuitry-based computational functions of electrical synapses

Two neurons connected via a gap junction are electrically coupled. This connection passes current between two cells according to their voltage difference. The difference may have both signs, positive and negative. So, the current is bidirectional. This electrotonic conduction is passive and very fast, almost instantaneous. Because it performs no amplification, energetically it is costless. Also, a gap junction needs no extra space for placing connexons in the opposed membranes of adjacent cells. This primitive analogue device is economically favoured and widely utilized in retinal neurocircuitry to establish various meaningful functions (Sterling and Laughlin, 2015). Four examples are below.

*Noise reduction*: Visual input pattern consists of numerous patches with various shapes and sizes. Intensities are similar within each patch. Signals of the patch carried by a group of neurons are mutually correlated but they are also superposed by noise components that are uncorrelated. The noise mostly originates in photon fluctuation, intracellular cascades, and synaptic transmissions. Noise components are random in both amplitude and phase. When all the neurons in that group are electrically coupled via gap junctions, the correlated components do not change much but the uncorrelated components efficiently cancel each other. Thus, gap junctions reduce noise (DeVries et al., 2002). In other words, they execute average pooling.
*Modulation between sensitivity and resolution*: Dopamine regulates electrical coupling between AII amacrine cells through a cAMP–mediated PKA cascade. The dopamine release from surrounding dopaminergic amacrine cells is modulated by light conditions. Consequently, the light conditions affect the coupling between AII amacrine cells. Under scotopic conditions, the AII-AII coupling area is so small as to gather only RBC synaptic inputs which diverged from a single photon-activated rod by avoiding noises from the surrounding inactive area, to increase absolute sensitivity. Under mesopic conditions, the AII-AII coupling area becomes larger to gather numerous RBC synaptic inputs which originate in many photon-activated rods to increase the contrast sensitivity of graded signal. Under photopic conditions, AII amacrine cells are involved with cone signals. The AII-AII coupling area becomes small again to increase resolution (Bloomfield and Volgyi, 2009).
*Frequency filtering*: Gap junction coupling in a 2D array of cone photoreceptors passes low spatial frequencies and attenuates high ones. Low spatial frequencies produce gradually changing voltage differences whereas high frequencies produce sharply changing voltage differences. Both conditions make the current flow slow and fast, respectively, through their membrane capacity (Cangiano and Asteriti, 2021).
*Synchronized activity*: Classical work in the retina revealed that neighboring retinal ganglion cells exhibit significant spike synchrony. The subsequent studies verified that gap junction–mediated electrical signals synchronize neural activity on millisecond timescales via cooperative interactions with chemical synapses (Trenholm and Awatramani, 1995; Trenholm et al., 2014).

## 7. Discussion

EM pictures of nervous tissues consist of sectional images of cell membranes and intracellular and extracellular spaces at various angles. We imagine 3D models of neurons in our brains by observing every morphological trait of EM images in the context of cell biology. Then, to express the result of our observation we trace cell contour lines and marked characteristic structures such as synapses. In this sense, we see EM pictures by our eyes but read EM pictures by our brains.

Seung explained how bad computers are at seeing edges, with the aid of a well-known illusion called the Kanizsa triangle (Seung, 2012). Briefly speaking, our mind can fill in the missing parts of the edges only when provided with the context of the other shapes. His explanation is helpful for us to consider our problem to reconstruct 3D neuronal circuitry. We interpret cell contour lines in conjunction with planar gradation on EM pictures. Experienced photographers prefer to use softly and adequately shaded pictures because shadings may sometimes contain more meaningful information than just lines. Annotation needs a high level of pattern recognition which we must cultivate over a long period of time. Such human pattern recognition may be still necessary even after advanced machine learning algorithms are introduced in computer-aided image analysis systems. Gap junctions seem to be especially challenging to handle smoothly even by the advanced connectomic inspection (Witvliet et al., 2021).

Connectomic studies on visual processing may deepen our understanding of the natural intelligence of pattern recognition (Sawant et al., 2023). In turn, that knowledge may contribute to the development of new algorithms of artificial intelligence and scaling up connectomic studies.

## Data availability statement

The original contributions presented in the study are included in the article material, further inquiries can be directed to the corresponding author.

## Ethics statement

The animal study was approved by Hyogo Medical University Committee on Animal Research. The study was conducted in accordance with the Act on Welfare and Management of Animals issued by the government of Japan.

## Author contributions

YT: Conceptualization, Data curation, Writing – original draft.
