# Corrigendum: Electrical synapses for a pooling layer of the convolutional neural network in retinas

**DOI:** 10.3389/fncel.2024.1404440

**Published:** 2024-04-22

**Authors:** Yoshihiko Tsukamoto

**Affiliations:** ^1^Department of Biology, Hyogo Medical University, Nishinomiya, Hyogo, Japan; ^2^Studio EM-Retina, Satonaka, Nishinomiya, Hyogo, Japan; ^3^Center for Systems Vision Science, Organization of Science and Technology, Ritsumeikan University, Kusatsu, Shiga, Japan

**Keywords:** gap junctions, ribbon synapses, electron microscopy, connectome, retinal neurocircuitry, rod pathway

In the published article, there was an error in section “**4. How CNN is compatible with the rod pathway**”, paragraph 4. In this paragraph, in the second sentence, the word “one” was incorrectly written as “on” and the duplicated word “dendrites” was erroneously included.

The corrected sentence appears below:

“The weight coefficients are mostly one but in a few cases two, where one RBC connects with one rod through two invaginating dendrites.”

The authors apologize for these errors and state that they do not change the scientific conclusions of the article in any way. The original article has been updated.

